# The effect of digital communication technology on older adults’ formal care use in Norway: a randomized controlled trial

**DOI:** 10.1186/s12877-026-07322-z

**Published:** 2026-04-11

**Authors:** Kristoffer Eikemo, Ira Malmberg-Heimonen, Erik B. Rasmussen, Björg S. A. Thordardottir, Anne Grete Tøge, Kjetil A. van der Wel

**Affiliations:** 1 Department of Social Work, Child Welfare and Social Policy, Oslo Metropolitan University, Oslo, Norway; 2 Department of Rehabilitation Science and Health Technology, Oslo Metropolitan University, Oslo, Norway; 3 Work Research Institute, Oslo Metropolitan University, Oslo, Norway

**Keywords:** Age in place, Survival analysis, Home and community-based care and services, Long-term care, Intervention study design/analysis

## Abstract

**Background:**

Norway’s population is ageing, increasing the pressure on already strained health and care services. In response, Norwegian policymakers have promoted a strategy of aging in place, i.e., that older adults are encouraged to live longer in their own homes. Low social contact is one of many risk factors for transitions to long-term care facilities. Digital technologies that facilitate social contact have been promoted as promising tools for increasing social contact among older adults and may also contribute to reduce strain on health and care services. In this RCT, we evaluate whether a digital communication technology, Komp, may prolong home-dwelling and reduce the need for formal care services among frail older adults. Given Komp’s documented potential to promote social connectedness and facilitate informal care, we hypothesize that Komp prolongs home-dwelling and reduces reliance on formal care services.

**Methods:**

We compared an intervention group offered to try Komp (*n* = 516) with a control group receiving services as usual (*n* = 595). 150 Komp units were delivered. Outcomes were assessed 28 months after randomization. We obtained administrative data from Statistics Norway and service use data from the boroughs’ journal records. We analyzed 1,099 participants (mean age: 84.6 years, range: 67–98; 64.7% [*n = 711*] female) using Cox proportional hazards regression (primary outcomes) and ZINB and ZIP regression models (secondary outcomes).

**Results:**

The intervention group had a higher, though non-significant, risk of moving to long-term care facilities compared to the control group (HR = 1.162 [0.944, 1.431]). ZINB regression models indicated that the intervention group experienced a reduction of five days of short-term institutional stays (IRR=0.833 [0.728, 0.952]). There were no differences between groups in the amount of other formal care services received.

**Discussion and implications:**

The implementation of Komp did not prolong home-dwelling among frail older adults. However, the results showed a reduction in formal care services, indicating that Komp may reduce some strain on health and care services. Future studies should evaluate the effect of other types of digital communication technologies on objective outcomes.

**Trial registration:**

ClinicalTrials.gov: NCT05919355. Date of registration: 16 June 2023.

**Supplementary Information:**

The online version contains supplementary material available at 10.1186/s12877-026-07322-z.

## Background

Like in all European countries, Norway’s population is ageing. By 2050, one in five Norwegians will be over the age of 70 [1], similar to projections across the OECD [2]. This demographic shift increases pressure on already strained health and care services. Norwegian policymakers have responded to the challenge by promoting a strategy of ‘ageing in place’, i.e. that older adults primarily should receive long-term care in their own homes rather than in long-term care facilities [3].

While ageing in place may promote independence and autonomy among many older adults [4], it is not feasible for all. The reasons why some older adults transition to long-term care are multifaceted and complex. Functional impairments and caregiver burden have been identified as key factors [5], along with low social contact [6]. Low social contact is further linked to lower quality of life [7, 8], cognitive decline [9, 10], and poorer overall physical health [5], which are further associated with greater reliance on formal care services [11]. Promoting social connectedness is therefore recognized as essential to ageing in place [12–14].

Digital communication technologies (DCTs) that allow for synchronous communication with formal caregivers, family, and friends [15], have been highlighted as promising tools to support healthy ageing [16, 17]. Previous research indicates that DCTs may enhance social connectedness, reduce loneliness, and improve well-being among older adults by facilitating communication with their social network [18–22]. However, despite promising findings, we lack studies with robust causal designs to evaluate their effects [23–27].

Many randomized controlled trials (RCTs) have evaluated DCT interventions among home-dwelling older adults on subjective outcomes such as loneliness and social isolation. However, most have focused on clinical content delivered through technology, such as telehealth [28] or cognitive therapy [29], rather than the social function of the intervention itself. To our knowledge, only two RCTs have examined DCTs as standalone interventions aimed at facilitating social connectedness among older adults. Czaja et al. [30] conducted a multisite trial in Florida and Georgia, United States, among individuals aged 65 or older living alone in independent housing. Participants received either PRISM, a computer system designed for older adults that included communication tools (e.g., photo sharing), or a control condition that provided similar information in a paper binder. Results showed that participants receiving PRISM experienced a significant increase in perceived social support and well-being, as well as a reduction in loneliness, compared to controls. In a follow-up RCT, Czaja et al. [31] evaluated PRISM 2.0, an updated tablet-based version with expanded social features, among individuals aged 65 years and above living in senior housing, rural locations, and assisted living communities in Florida and Georgia. The intervention group was compared to a control group using a tablet with standard applications. Both the PRISM 2.0 and standard tablet groups living in senior housing and rural areas reported reductions in loneliness and social isolation and improvements in quality of life and social support. To the best of our knowledge, no RCT-studies has investigated the effect of the social function of DCTs on objective service outcomes, such as formal care use and transitions to long-term care.

The present study addresses these research gaps by evaluating the effect of the DCT device *Komp* on formal care use among home-dwelling older adults aged 67 years and above. Komp was designed for individuals who are unfamiliar with, or struggle with, conventional digital technologies [32]. All interactions beyond turning the device on or off and adjusting the volume are managed by the user’s selected social network through a connected app. This network can initiate video calls and send photos and text messages, which appear automatically on the Komp screen. Findings from previous research on Komp indicate that this tool does increase feelings of safety, supports meaningful digital contact between users and their social network, and thereby facilitates emotional care [15, 32–35].

**Fig. 1:**
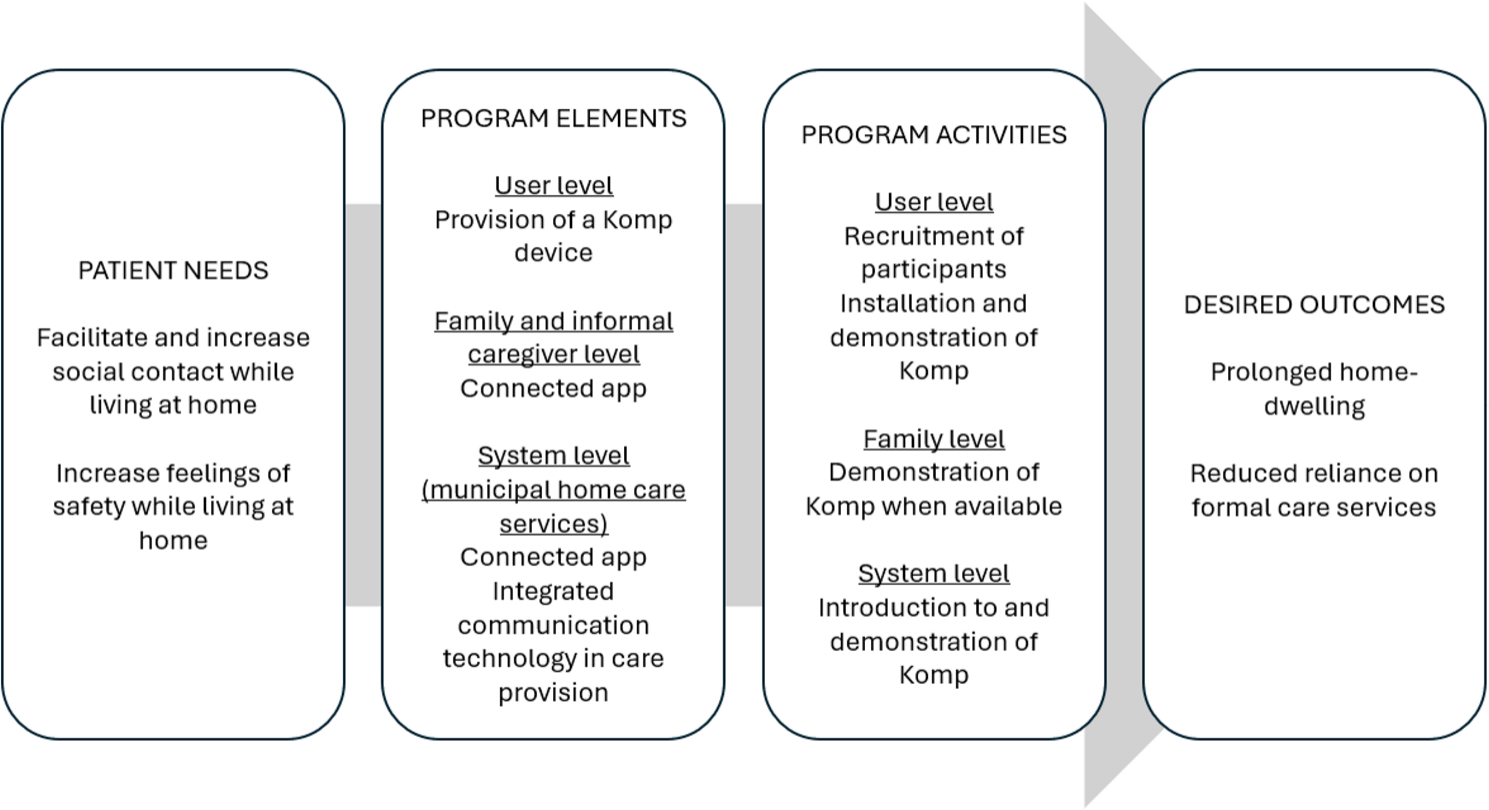
Logic model (based on the pipeline-model [36])

Based on the indications that Komp supports social contact and increases feelings of safety, our trial’s program theory proposes the following mechanisms. First, increased social contact and perceived safety may mitigate factors such as loneliness and insecurity that increase the likelihood of moving to a long-term care facility and greater service needs. Second, Komp may delay transitions to long-term care and reduce reliance on home-based services by facilitating informal care. Such informal care may be emotional support and everyday reassurance, and increased digital contact could make it easier for informal carers to identify emerging needs and provide timely support. Thus, although not providing clinical content, we hypothesize that Komp prolongs home-dwelling and reduces reliance on formal care services among older adults (Fig. 1).

In accordance with the trial protocol (NCT05919355), our primary objective is to assess whether access to Komp prolongs service recipients’ home-dwelling by delaying their move to long-term care facilities. Our secondary objective is to evaluate whether access to Komp reduces the use of other formal care services: home nursing care, short-term institutional stays, practical assistance, and safety alarms. The original implementation plan also included municipal care staff’s use of Komp, which ultimately did not occur due to delays. This deviation has implications for our program theory and expected outcomes. While, according to our program theory, the intervention’s ability to address social needs and strengthen informal care should still influence the use of formal services, the anticipated effects may be weaker when the device is not simultaneously adopted by formal care providers.

## Methods

### Study design

This study is designed as a field trial with a parallel-group assignment and an intention-to-treat (ITT) approach. It is conducted as part of the BoVel project – a collaboration between Oslo Metropolitan University, Oslo Municipality, and Abilia, which is a private company that develops and sells Komp. Recruitment began on December 21, 2022, with data collection spanning from October 13, 2022, to March 1, 2025. Our reporting follows the CONSORT checklist [37] as far as applicable.

### Study context

Our study was conducted in three boroughs of Oslo, Norway: Nordstrand, St. Hanshaugen, and Østensjø. In international comparison, Norway has a comprehensive welfare state with redistributive benefits and universal health services, including long-term care services for older adults [38]. Long-term care is largely a public responsibility, with municipalities covering 90% of the costs [39]. Many municipalities in Norway, including Oslo, have waiting lists for long-term care facilities [40], making home-based services, such as home nursing care, the primary means of supporting older adults in need of care [41]. Nursing homes are generally reserved for those with the most complex needs, as reflected in the fact that the average length of stay in a Norwegian nursing home is approximately two years [42].

### Participants

**Fig. 2:**
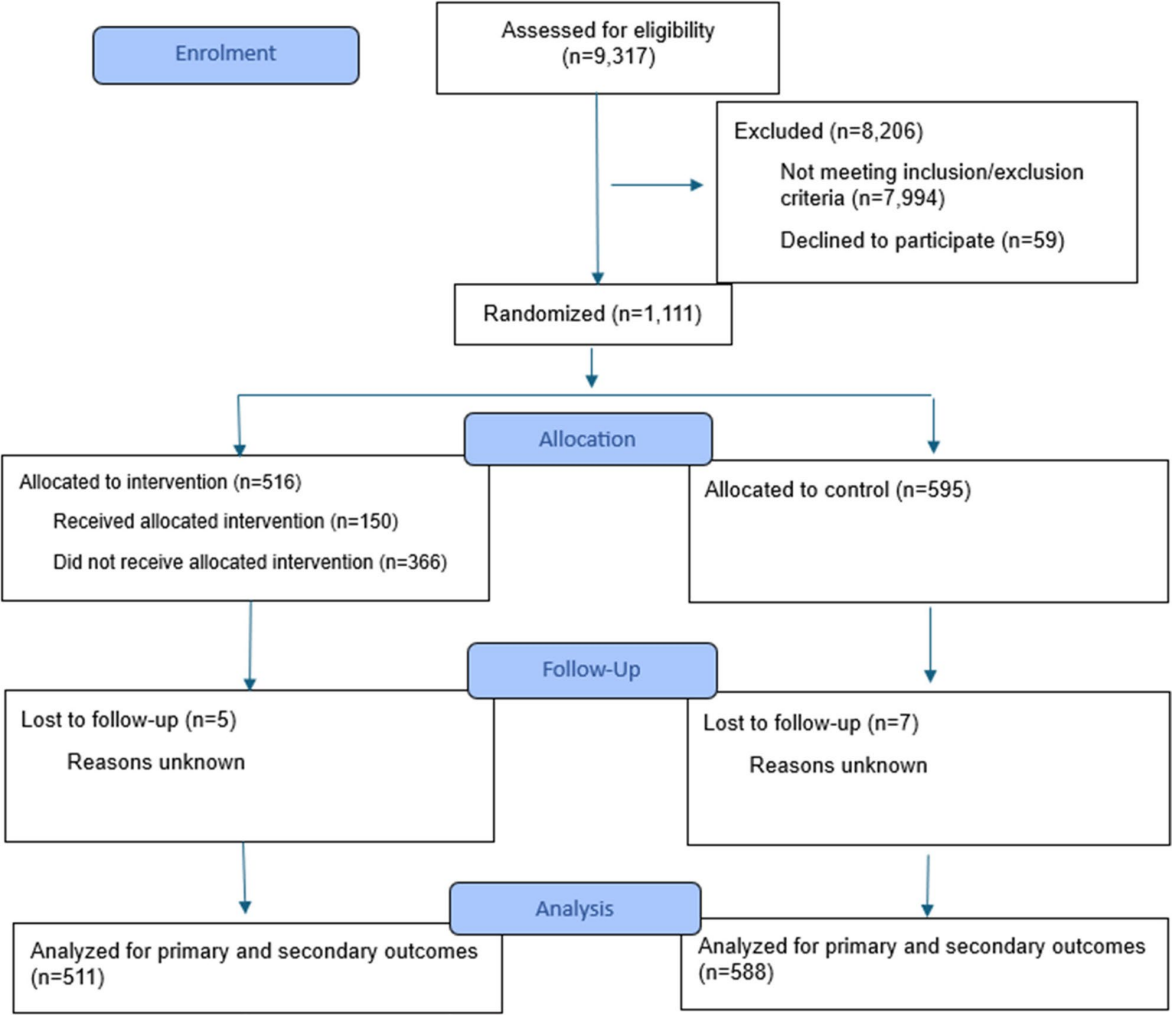
CONSORT 2025 flow diagram [37]

We identified potential participants among 9,317 municipal health and care service records from three Oslo boroughs. Eligible participants had to (i) be 67 years or older; (ii) dwell in a private home (i.e., not in a permanent care or nursing home); (iii) have a registered address in one of three participating boroughs of Oslo, Norway; and (iv) be registered recipients of municipal home nursing care. Individuals were excluded if they had full function in all three of the following Activities of Daily Living (ADL) measures, as assessed by the services: outdoor mobility, memory, and cooking. They were excluded because having full function in all three areas meant a very low risk of moving to long-term care during the trial, and thus, they were outside the intervention target group. This exclusion process resulted in 1,170 eligible participants. Written information about the project was sent, with the option to decline participation. Information was first distributed electronically, followed by a postal version if the electronic version was not opened within 14 days. In total, 59 individuals declined participation, leaving 1,111 participants. Fig. 2 demonstrates the flow chart for the study.

### Assignment to intervention and control groups

In RCTs, eligible participants must have a defined and equal probability of being assigned to the intervention to create groups that are comparable with respect to both measured and unmeasured characteristics [43]. In this study, eligible participants (*n* = 1,111) were identified from municipal records as of December 21, 2022, with the data backdated to October 13, 2022. To ensure equal probability of allocation, we applied a form of permuted block randomization [43], treating each of the three boroughs as a separate block. Separate lists were created for each borough, and participants were randomly ordered by generating random number sequences in Stata. Local project coordinators offered Komp to names on the randomly ordered lists, working from the top down. We had 60 Komps available in St. Hanshaugen, and 120 in each of the two larger boroughs. Because many participants were unwilling to receive Komp or were unreachable during the delivery period, we reached half the lists and stopped the offers before all available Komps (*n* = 300) had been distributed. At this point, we had made an offer to 32% of the 1,111 participants.

When persons in the intervention group did not receive Komp, we recorded them as no-shows. In accordance with the ITT approach, we kept them (*n* = 157) in the intervention group, as the control group would also include unavailable individuals. This resulted in 516 participants in the intervention group and 595 in the control group. Blinding was not possible due to the nature of the intervention. Although we cannot rule out the possibility that knowledge of group assignment influenced how home care services were delivered, we have no indications that this occurred. Qualitative interviews with home care professionals do not suggest any systematic differences in follow-up or service provisions [32], and any such differences would likely have been detected.

After participants were offered Komp, they had about 14 days to accept. Those who accepted (*n* = 150) received a Komp device within one week, which they could use freely. Installation was carried out by project coordinators or other trained staff, and participants received written information about technical support. In most cases, the offer was made in person, accompanied by a demonstration of the device, and, when possible, with relatives present. Most devices were delivered over a period of 10 months, except for two cases receiving Komp 21 months after distribution commenced. At any time, participants who had received a Komp could stop and return it to the municipality. Twelve participants returned the device earlier, after, on average, using it for 8.5 months. Participants who declined the offer continued to receive services as usual (*n* = 209). In accordance with the ITT approach, all individuals offered Komp were included in the intervention group. Control group members continued to receive home care services as usual.

### Intervention

Komp is a DCT developed by the Norwegian company No Isolation to support social contact between older adults and their social network. It was developed using a user experience design, including interviews with professionals within gerontology and with older adults who experienced difficulties using conventional digital technologies. The development process included concept workshops and pilot testing with members of the target group. The device consists of a screen with a single button for turning it on and off, as well as adjusting the volume. The user’s social network uses a connected app to send photos and messages and make video calls. Calls are automatically connected after ten seconds unless the user turns the device off. While off, no calls go through, but a small blinking light indicates incoming calls. An important clarification is that the Komp user cannot initiate contact through the Komp screen; they can only receive. The design is intentionally designed to be suitable for individuals with limited technological competence. However, it also limits opportunities for interaction, making Komp valuable only if others actively engage. This has, in some instances, been experienced as disempowering [32]. At the same time, previous studies have indicated that users develop compensatory strategies, such as using the telephone to request contact via Komp when they wish to connect [44]. While the simplified interface may be too simple for some, it lowers the technological threshold for participation and may therefore include individuals who might otherwise be excluded from this kind of digital communication. See Akhtar [33] and Rasmussen et al. [32] for more information on Komp.

### Success of randomization

We estimated significance using two-sided T-tests for continuous variables and chi-squared tests for categorical variables (Table 1). The results show that the groups are balanced, except for wealth and marital status: Participants in the intervention group had slightly higher wealth, while a higher proportion of participants in the control group were married. We adjust for these differences in the effect analyses.

**Table 1:** Baseline demographics

	Control group (*n* = 595)	Intervention group (*n* = 516)	Difference (*p*-value)
Age	84.36 (7.76)	84.77 (7.74)	0.411 (0.378)
Female	0.642 (0.480)	0.651 (0.477)	0.009 (0.702)
Borough			
St. Hanshaugen	0.187 (0.390)	0.205 (0.404)	0.019 (0.429)
Nordstrand	0.415 (0.493)	0.376 (0.485)	0.039 (0.183)
Østensjø	0.398 (0.490)	0.419 (0.494)	0.020 (0.493)
Educational level			
Low	0.242 (0.429)	0.246 (0.429)	0.004 (0.874)
Medium	0.496 (0.500)	0.494 (0.500)	0.002 (0.957)
High	0.242 (0.429)	0.240 (0.428)	0.002 (0.947)
No or unreported	0.020 (0.141)	0.019 (0.139)	0.001 (0.925)
Income decile (EU standard)	2.56 (2.15)	2.68 (2.10)	0.115 (0.367)
Wealth decile (EU standard)	6.15 (2.77)	6.50 (2.62)	0.349 (0.033)**
Living alone	0.699 (0.459)	0.713 (0.453)	0.027 (0.609)
Immigrant	0.081 (0.273)	0.085 (0.280)	0.005 (0.780)
Number of children			
0	0.089 (0.285)	0.085 (0.280)	0.004 (0.823)
1	0.165 (0.371)	0.165 (0.371)	0.000 (0.999)
2	0.434 (0.496)	0.399 (0.490)	0.034 (0.246)
3	0.173 (0.379)	0.203 (0.403)	0.030 (0.195)
4+	0.071 (0.256)	0.062 (0.241)	0.009 (0.568)
No information	0.069 (0.254)	0.085 (0.280)	0.016 (0.307)
Marital status			
Married	0.247 (0.432)	0.198 (0.399)	0.049 (0.049)**
Never married	0.099 (0.299)	0.114 (0.319)	0.015 (0.413)
Widow/widower	0.440 (0.497)	0.452 (0.498)	0.011 (0.708)
Divorced/separated	0.213 (0.410)	0.236 (0.425)	0.023 (0.359)
ADL-score	2.612 (0.747)	2.649 (0.772)	0.037 (0.418)

Figures for control and intervention groups are mean (SD) | *p < 0.1; **p < 0.05; ***p < 0.001

### Measures

#### Primary outcome

The primary outcome was time spent living at home, measured in days from the start of the project (October 13, 2022) until either moving to a long-term care facility or the end of the study period (March 1, 2025).

##### Time spent living at home

Data was obtained from Gerica. Gerica is an electronic patient journal and administrative system that contains information on service recipients within the municipal health sector. The data is collected by municipal care workers and professionals. The moving date was defined as the start date of the service “long-term institutional stay”. Participants who died at home were coded in three different ways in the analyses: they were (i) censored at the date of death, (ii) treated as events in the same way as moving to a long-term care facility, and (iii) treated as a competing event (see statistical analysis section). Those who remained living at home throughout the observation period were censored at the study end date. Two participants in the control group had missing data on service start.

#### Secondary outcomes

Secondary outcomes were measured as the amount and type of care services received.

##### Home nursing care

We obtained data on duration and weekly hours of home nursing care decisions from Gerica. These were operationalized as total hours per participant over the project period. As receiving home nursing care was an inclusion criterion, almost all participants had a registered value. Some (*n* = 24) who discontinued early were assigned zero hours.

#### Short-term institutional stays

Short-term institutions are time-limited services intended for rehabilitation, assessment of care needs, or respite for family caregivers. The aim is usually for older adults to return home after illness, hospitalization, or a period of increased care needs. These were recorded as whole days in Gerica, with no value for intensity. We coded stays as (i) total number of days in a short-term institution, (ii) total number of days capped at 200 to handle extreme outliers, and (iii) total number of unique stays at a short-term care facility. Participants without any records were assigned a value of zero.

#### Practical assistance

Practical assistance includes home support services, aimed at helping individuals manage daily activities such as financial management and general routines. Recorded with duration and weekly hours in Gerica, from which we calculated the total hours received per participant throughout the project period. We added an outcome variable capped at 100 h to handle outliers. Participants without records were assigned a value of zero.

#### Safety alarms

Safety alarms allow older adults to call for assistance at any time of day, in case of a fall or a sudden health problem. We obtained data on safety alarm activations from the supplier Careium. Each activation was coded as a unique observation in the data set, with a reason for activation labeled by healthcare professionals. We excluded records labeled as test. Participants with no records were assigned a value of zero.

### Statistical power

**Fig. 3:**
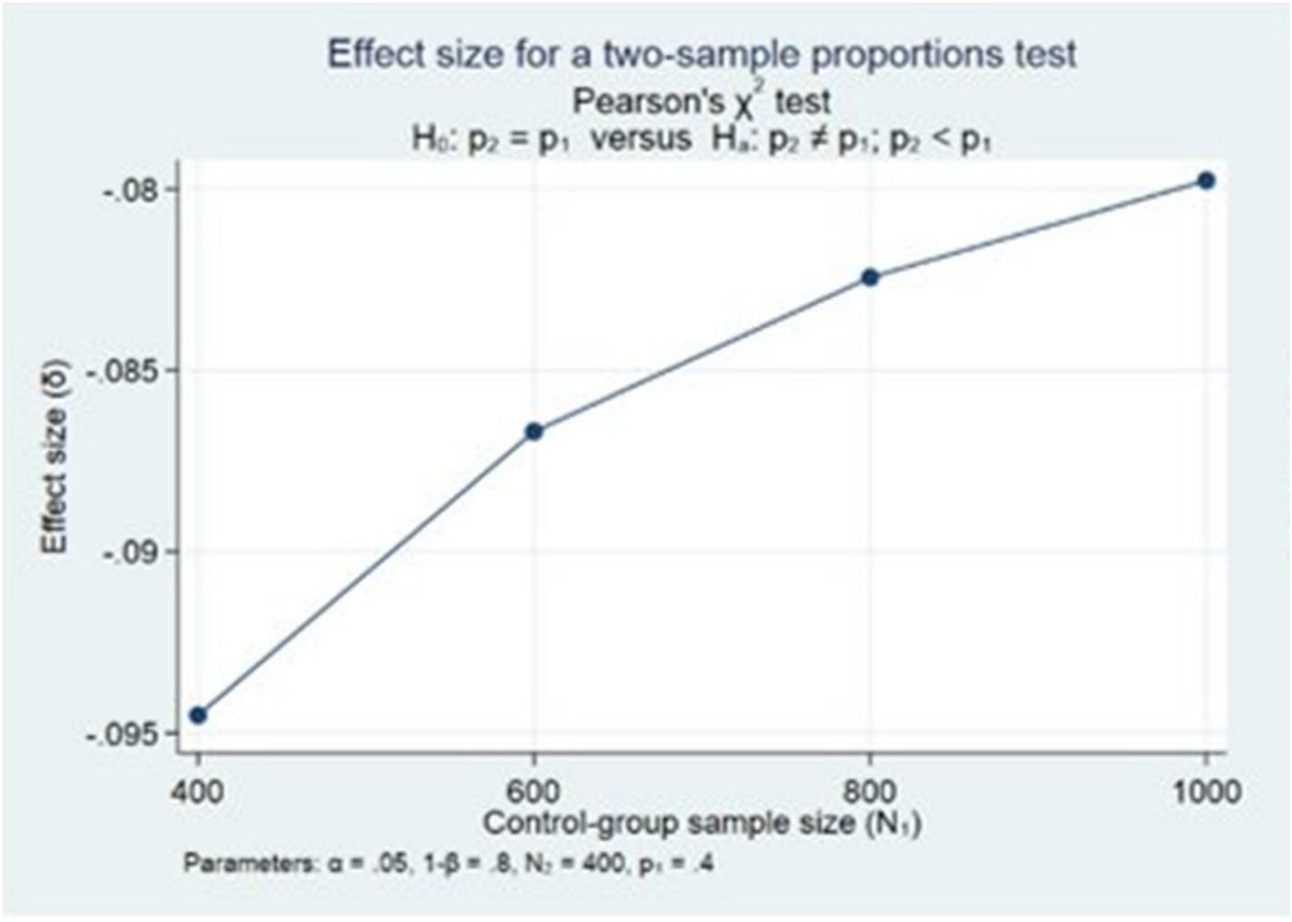
Statistical power calculations for primary outcome

We conducted power calculations prior to the trial (Fig. 3) using a two-sample proportions test in Stata (StataCorp, 2023). Following conventional thresholds of a 5% significance level and 80% power, we estimated that approximately 400 participants per group would be required to detect a 10-percentage point difference in the primary outcome.

However, the realized exposure differed from these assumptions. Although 516 participants were allocated to the intervention group, only 150 received a device, and a much smaller proportion used it meaningfully. This reduces the statistical power of the trial and must be accounted for when interpreting the results.

### Statistical analysis

All statistical analyses were conducted using Stata 18 [45]. For the primary outcome, time until moving to a long-term care facility, we used survival analysis. Hazard ratios (HRs) were first estimated in a crude model, before adjusting for baseline differences using the Cox proportional hazards model [46]. Here, HRs above 1 indicate a higher risk of moving, and HRs below 1 indicate a lower risk. We tested the proportional hazards assumption using Schoenfeld residuals [47]. No violations were detected. Further, we had a censoring issue. The Cox model assumes non-informative censoring, meaning that participants who are censored should drop out for reasons unrelated to the outcome [48]. In our case, deaths are directly related to the risk of moving to a long-term care facility. Thus, by censoring deaths, we violate this assumption. However, if we treat deaths as events of interest, we measure an outcome different from our primary outcome. For robustness, we conducted two alternative analyses treating deaths as both censored and events. In addition, we applied a competing-risks model [49] to account for deaths as a competing event, reporting subhazard ratios (SHRs).

All secondary outcome variables were treated as count data. Almost all of them had issues with overdispersion (see Table 3), which violates the Poisson regression assumption of equal mean and variance [50]. To account for this, we used negative binomial regression models for home nursing care. The remaining secondary variables had, in addition, an excess of zero values. To address both overdispersion and excess zeros, we applied zero-inflated negative binomial (ZINB) regression models for days of short-term institutional stays, practical assistance, and safety alarm activations. ZINB combines a logistic component, which estimates the probability of structural zeros, with a negative binomial component, which models the count distribution among service users [51]. For the variable representing unique number of short-term stays, overdispersion was not significant. Although the variance was higher than the mean, the alpha parameter in the ZINB model was not significantly different from zero (not shown), indicating that overdispersion was not an issue [52]. Therefore, we used a zero-inflated Poisson (ZIP) model for this outcome.

Model comparisons using AIC and BIC favored negative binomial models over Poisson for all secondary outcome variables [53]. Results are reported as incidence rate ratios (IRRs) for the count component, and odds ratios (ORs) for the excess zero component.

## Results

In Table 2, we present descriptives for the primary and secondary outcomes. Over the 870-day follow-up, 45.9% of participants remained at home, 32.5% transitioned to long-term care, and 21.7% died while living at home. Rates of moving to care facilities were highest in the intervention group, while the control group had more deaths and more participants staying at home throughout the period. In total, the population accumulated 651,158 days of living at home, corresponding to an incidence rate of 0.0005. For all secondary outcome variables, the variance exceeds the mean, indicating overdispersion [52].

**Table 2:** Descriptive statistics for primary and secondary outcomes

	**Control group (*n*** **= 586)**		***Intervention group (n = 511)***		**Total (*n*** **= 1,097)**		
**Primary outcome descriptives**							
	**Mean (st.dev.)**		***Mean (st.dev.)***		**Mean (st.dev.)**		**Min-max**
Moved to care facility	0.311 (0.463)		*0.341 (0.474)*		0.325 (0.468)		0–1
Died at home	0.222 (0.416)		*0.211 (0.409)*		0.217 (0.412)		0–1
Stayed at home	0.468 (0.499)		*0.448 (0.498)*		0.459 (0.499)		0–1
Days until moving	609.54 (304.42)		*575.28 (324.94)*		593.58 (314.47)		2-870
Total time at risk	357,188		*293*,*970*		651,158		
Incidence rate	0.0005095		*0.0005919*		0.0005467		
**Secondary outcome descriptives**							
	**Mean (std.dev.)**	**Variance**	***Mean (std.dev.)***	***Variance***	**Mean (std.dev.)**	**Variance**	**Min-max**
Home nursing care							
Total number of hours	412.10 (497.74)	247748.9	*415.80 (491.08)*	*241157.7*	413.82 (494.43)	244465.2	0-5440.1
Short-term institutional care							
Non-zero value	0.541 (0.499)	0.249	*0.542 (0.499)*	*0.249*	0.541 (0.499)	0.249	0–1
Total number of days	31.0 (55.30)	3057.6	*25.90 (39.48)*	*1558.8*	28.63 (48.63)	2365.1	0-684
Total number of days (max 200)	29.01 (41.35)	1709.7	*25.56 (37.74)*	*1424.4*	27.40 (39.73)	1578.6	0-200
Unique number of stays	1.112 (1.880)	3.534	*1.047 (1.908)*	*3.641*	1.082 (1.893)	3.582	0–20
Practical assistance							
Non-zero value	0.687 (0.464)	0.215	*0.665 (0.472)*	*0.223*	0.677 (0.468)	0.219	0–1
Total number of hours	32.31(39.76)	1580.8	*31.80 (60.32)*	*3638.3*	32.08 (50.35)	2535.1	0-739.31
Total number of hours (max 100)	30.20 (31.51)	992.70	*26.80 (31.26)*	*977.20*	28.62 (31.42)	987.48	0-100
Safety alarm							
Non-zero value	0.257 (0.437)	0.191	*0.266 (0.442)*	*0.196*	0.261 (0.439)	0.193	0–1
Number of activations	7.660 (29.33)	859.99	*8.959 (51.63)*	*2665.2*	8.264 (41.21)	1698.1	0-1089

Figures in italics represent the intervention group

In Table 3, we present the results for the primary outcome, along with HRs from the Cox (1–4) and Fine-Gray models (5–6). Here, we assess the time to move into a long-term care facility. Across all models, the estimated HRs for the intervention group range from 1.090 to 1.162. This suggests that the intervention group had approximately a 10% higher risk of moving to a long-term care facility than the control group. However, the differences between the intervention and control groups are not statistically significant.

**Table 3:** Days until moving to a long-term care facility. Cox proportional hazards model (1–4) and Fine-Gray model (5–6). Reported as HRs (95% CI) and SHRs (95% CI)

	Model 1 (HR) *N* = 1,097	Model 2 (HR) *N* = 1,092	Model 3 (HR) *N* = 1,097	Model 4 (HR) *N* = 1,092	Model 5 (SHR) *N* = 1,097	Model 6 (SHR) *N* = 1,092
Intervention	1.162 (0.944, 1.431)	1.126 (0.914, 1.388)	1.097 (0.934, 1.289)	1.090 (0.926, 1.282)	1.136 (0.923, 1.398)	1.100 (0.891, 1.357)
Wealth		1.079*** (1.034, 1.126)		1.042** (1.010, 1.075)		1.080*** (1.037, 1.125)
Married		1.086 (0.844, 1.397)		1.273** (1.056, 1.534)		0.978 (0.762, 1.254)

Deaths right censored (1, 2); Deaths treated as event (3, 4); Deaths as competing event (5, 6) | *p < 0.1; **p < 0.05; ***p < 0.001

Implementation data show that participants who had registered use on their Komp were, on average, registered for 1.1 activities per week (video calls, images, or messages). Ten participants had fewer than ten interactions in total, and 23 had fewer than 0.5 activities per week. 20 participants never received video calls, but only images and messages (46 activations on average among these).

In Table 4, we present IRRs and ORs from models assessing group differences in formal care service use. Models 7 and 8 estimate total hours of home nursing care using negative binomial regression. Both models show no significant differences between groups. The estimated alpha parameter confirms overdispersion, as it is significantly different from zero [52], supporting the model choice.

**Table 4:** Secondary outcomes from negative binomial regression models (7, 8), ZINB regression models (9–12, 15–18), and ZIP regression models (13–14). IRRs (95% CI) and log-odds (95% CI)

	Hours of home nursing care		Days of short-term institutional stays				Number of unique short-term institutional stays		Hours of practical assistance				Safety alarm activations	
	Uncapped		Uncapped		Capped		Uncapped		Uncapped		Capped		Uncapped	
	Model 7 (IRR) *N* = 1,099	Model 8 (IRR) *N* = 1,094	Model 9 (IRR) *N* = 1,099	Model 10 (IRR) *N* = 1,094	Model 11 (IRR) *N* = 1,099	Model 12 (IRR) *N* = 1,094	Model 13 (IRR) *N* = 1,099	Model 14 (IRR) *N* = 1,094	Model 15 (IRR) *N* = 1,099	Model 16 (IRR) *N* = 1,094	Model 17 (IRR) *N* = 1,099	Model 18 (IRR) *N* = 1,094	Model 19 (IRR) *N* = 1,099	Model 20 (IRR) *N* = 1,094
Count component														
Intervention	1.009 (0.874, 1.165)	1.012 (0.875, 1.170)	0.833** (0.728, 0.952)	0.828** (0.725, 0.946)	0.879** (0.774, 0.998)	0.869** (0.765, 0.987)	0.899 (0.775, 1.044)	0.923 (0.800, 1.065)	1.015 (0.885, 1.164)	1.014 (0.885, 1.160)	0.914 (0.809, 1.032)	0.921 (0.815, 1.040)	1.141 (0.813, 1.602)	1.080 (0.772, 1.513)
Wealth		0.994 (0.968, 1.020)		1.018 (0.993, 1.044)		1.016 (0.992, 1.040)		1.021 (0.992, 1.050)		0.958** (0.933, 0.983)		0.979* (0.95, 1.002)		0.945 (0.883, 1.012)
Married		0.930 (0.782, 1.106)		1.414*** (1.213, 1.649)		1.274** (1.100, 1.476)		2.277*** (1.964, 2.640)		0.716** (0.582, 0.881)		0.787** (0.654, 0.947)		0.587** (0.365, 0.945)
Constant	412.10*** (373.57, 454.61)	435.52*** (360.62, 525.97)	57.07*** (52.09, 62.53)	46.32*** (38.61, 55.57)	53.47*** (49.03, 58.32)	45.66*** (38.38, 54.33)	1.669*** (1.513, 1.841)	1.084 (0.871, 1.347)	46.72*** (42.58, 51.27)	63.20*** (52.59, 75.97)	44.00*** (40.53, 47.77)	51.51*** (43.74, 60.66)	24.67*** (19.21, 31.68)	39.29*** (23.10, 66.83)
Excess zero component^a^														
Intervention			0.992 (0.781, 1.260)	0.992 (0.780, 1.263)	0.993 (0.782, 1.261)	0.993 (0.781, 1.264)	0.866 (0.582, 1.288)	0.856 (0.545, 1.345)	1.101 (0.849, 1.428)	1.206 (0.911, 1.597)	1.096 (0.848, 1.416)	1.200 (0.910, 1.581)	0.965 (0.720, 1.294)	0.997 (0.740, 1.343)
Wealth				0.973 (0.931, 1.017)		0.973 (0.931, 1.017)		0.985 (0.904, 1.073)		1.013 (0.961, 1.067)		1.014 (0.963, 1.068)		0.931** (0.880, 0.986)
Married				0.787 (0.589, 1.053)		0.786 (0.588, 1.051)		1.864 (1.185, 2.933)		5.725*** (4.188, 7.826)		5.661*** (4.154, 7.715)		1.928** (1.298, 2.861)
Constant			0.841** (0.715, 0.992)	1.048 (0.755, 1.453)	0.844** (0.717, 0.993)	1.051 (0.758, 1.456)	0.501*** (0.390, 0.643)	0.368 (0.191, 0.708)	0.446*** (0.373, 0.534)	0.259*** (0.170, 0.372)	0.457*** (0.383, 0.545)	0.256*** (0.174, 0.377)	2.220*** (1.746, 2.824)	3.059*** (1.992, 4.697)
Alpha	1.473 (1.368, 1.585)	1.463 (1.36, 1.57)	0.671 (0.597, 0.754)	0.648 (0.577, 0.729)	0.603 (0.536, 0.677)	0.592 (0.527, 0.665)			0.872 (0.780, 0.976)	0.845 (0.755, 0.945)	0.680 (0.609, 0.760)	0.669 (0.599, 0.747)	2.316 (1.723, 3.112)	2.225 (1.665, 2.974)

^a^Estimates in excess zero components are reported as odds ratios | Dependent variable with no cap (7–10, 13–16, 19–20). Dependent variable with 200 days cap (11, 12); Dependent variable with 100 h cap (17, 18) | * *p* < 0.1; **p < 0.05; ***p < 0.001

Models 9–12 report results from the ZINB models for short-term institutional stays. We find a significant reduction in the total number of days spent in short-term care for the intervention group (model 9: IRR = 0.833 [0.728, 0.952]) of approximately 17%. Given the control group average of 31 days, this translates to a reduction of approximately five days per participant. The adjusted model (10) shows similar results (IRR = 0.828 [0.725, 0.946]). The findings remain consistent when the dependent variable is capped at 200 days (models 11 and 12). The absence of significant effects in the excess zero component suggests that the odds of receiving the service were similar between groups.

In models 13 and 14, we present results from the ZIP models for the number of unique short-term institutional stays, showing no differences between groups. Similarly, the ZINB models assessing hours of practical assistance (models 15–18) and the number of safety alarm activations (models 19–20) show no significant differences between groups.

We conducted two post hoc analyses (not shown) to examine treatment effects among the treated: an *as-treated* analysis, comparing participants who received a Komp with controls, and a *per-protocol* analysis, comparing those with registered Komp use with controls (Ahn & Kang, 2023). For the primary outcome, the as-treated analysis was largely consistent with the main analysis but indicated a significantly lower risk of moving to long-term institutional care and dying at home in the analyses treating deaths as an event (HR = 0.692 [0.532, 0.901]). In contrast, the per-protocol analysis consistently indicated that Komp users had a significantly lower risk of moving to long-term institutional care (HR = 0.255 [0.113–0.575]). For the secondary outcomes, both post hoc analyses showed that the treated used formal care services more often. Significant effects were observed for home nursing care (as-treated IRR = 1.396 [1.132, 1.720]), number of short-term institutional care stays (as-treated IRR = 1.244 [1.025, 1.509]; per-protocol IRR = 1.447 [1.068, 1.960]), practical assistance (as-treated IRR = 1.400 [1.181, 1.659]; per-protocol IRR = 1.682 [1.300, 2.176]), and safety alarm activations (as-treated IRR = 1.741 [1.101, 2.752]). No significant effects were observed for the number of days in short-term institutional stays.

## Discussion

This trial investigated the effects of Komp on time spent living at home and on the use of formal care services among older adults receiving long-term home care. The findings did not support our hypothesis that access to Komp would prolong home-dwelling. On the contrary, the intervention group had a 10 to 16% higher risk of moving to a long-term care facility. These findings, however, were not statistically significant. Given the reduced statistical power, these estimates cannot be taken as evidence of a true effect.

Still, the direction and consistency of the estimates across model specifications warrant some considerations. Although speculative, we propose two mechanisms for consideration. First, enhanced digital contact through Komp may have enabled closer monitoring of the participants’ health status, leading family members or other informal caregivers to advocate for continued care in long-term care facilities. Another interpretation is that unintended harms need to be considered. For example, if digital contact substituted higher-quality in-person interactions, the intervention may have weakened rather than strengthened social connectedness. However, previous studies of Komp indicate that its use does not replace other forms of social contact nor reduce satisfaction with social relationships [33, 34], making the latter explanation less likely.

While the lack of significant effects in the primary outcome may reflect no intervention effect, the pattern may also reflect limited statistical power and implementation constraints. Due to delays in approval processes, the trial commenced six months later than planned, by which time several participants had become unavailable. As a result, only half of the intended devices were distributed, reducing the contrast between the groups. This reduced the trial’s statistical power, increasing the risk that actual effects would go undetected.

In addition, the intervention was implemented in a narrower form than initially intended. The trial design intended for dual use of Komp, by both family members and municipal care staff. Due to the change in the implementation plan, the municipal care services never adopted the use of Komp. Therefore, we could measure only the effect of use from the participants’ private social network, which previous research has shown to be mainly family use [32]. This likely limits the effectiveness of the intervention and the contrast between groups, as it may have led to fewer participants receiving contact through Komp. On the other hand, the absence of use from the health care services allowed us to isolate the effect of family use alone, and thus avoid conflating effects.

Further, the results show that the intervention group experienced reduced usage of some formal care services, as we recorded a significant reduction in the duration of short-term institutional stays. On average, the intervention group spent approximately five fewer days in short-term care compared to controls. Short-term care is primarily intended for rehabilitation after surgery, medical assessment, or temporary relief for informal caregivers. It is possible that increased social contact has supported the recovery process, as Komp users could bring their devices to the short-term care facilities. Empirical evidence indicates that access to a social network and perceived support are associated with postoperative pain and anxiety and the length of stay after major operations [54] and that older adults may overestimate the social support they receive, often leading to an unmet need for help in activities of daily living [55]. Such activities involve everyday tasks that may be supported remotely by family members. The assurance of continued social contact after discharge may also have made it easier for participants to return home. We must also consider the possibility that Komp facilitated informal caregiving by enabling family members to provide practical or emotional support remotely. Reduced caregiver burden may therefore have reduced the need for respite, thereby contributing to shorter institutional stays among the participants. On the other hand, the facilitated contact during the short-term stay might have allowed family members to monitor the users’ condition more closely. If so, they may have been more likely to advocate for long-term institutional care after admission to short-term care. The lack of data on in-person visits during the admission period limits the interpretation of these findings.

Notably, there were no differences between groups in hours of home nursing care, number of short-term institutional stays, hours of practical assistance, or safety alarm activations. This pattern suggests that the reduction in duration of short-term institutional stays was in fact a true reduction and not offset by increased use of other formal home-based services. However, as the intervention group showed a slight increase in long-term institutional stays, we cannot rule out that some substitution occurred between short-term and long-term care. The risk that the observed reduction in short-term institutional stays may be significant at random must also be considered. Thus, the findings must be interpreted with caution.

Our post hoc analyses complicate the interpretation of our results. We performed an *as-treated* analysis comparing those who received a Komp with controls, and a *per-protocol* analysis comparing participants with recorded Komp use to controls. In these analyses, we are no longer following a randomized design. Although they may reflect a true effect of Komp, they may also be due to selection bias. One possible explanation for the results is that frailer participants were more likely to accept the offer, as more active and technologically competent individuals have previously rejected Komp because they perceived it as too simplistic or even disempowering [32]. This could explain the higher use of home-based formal care services that we observed in both post hoc analyses. On the other hand, these findings could come as a consequence of the prolonged home-dwelling observed in the per-protocol analyses. Yet, the observed prolonged home-dwelling itself could be a result of selection bias, as participants with the most active family ties would be more likely to have registered Komp activity and to receive more informal support, as previous studies have shown [35, 44]. Overall, these findings are best interpreted as results of selection bias rather than causal effects, and we therefore place greater weight on the ITT analyses. Still, we need more robust evidence to confidently conclude our findings.

### The risk of unintended harm

Although Komp was designed to enhance social connectedness and thus reduce reliance on formal care services, we cannot rule out the possibility of unintended harms. For example, 97 Komp devices were not used during the project period. This means that 97 participants accepted the offer, but did not have anyone reach out through the connected app. In some of these cases, participants might have paid little attention to it, and no harm may have occurred. However, for others, the presence of an unused device could have acted as a constant reminder of limited social contact. One could, in this scenario, imagine that the device reinforced feelings of social isolation and thus induced harm to participants in the intervention group. In addition, the original implementation plan included more active involvement from municipal home care services, meaning that communication through Komp was not intended to rely solely on participants’ private social networks. If participants accepted Komp with an expectation that it would facilitate social contact despite not having a significant social network, it is possible that the deviation from the original plan may have caused harm to the participants. One potential way to prevent this would have been to select participants who were more closely aligned with Komp’s intended target group. For example, only including individuals with digitally active family members, only those with family members living outside of the municipality, or participants identified by the municipal home care services as suitable candidates. Such an approach, however, has downsides, as it could exclude individuals who might otherwise benefit from the intervention, thereby creating a double burden for those already at risk of being left out.

### Findings in the context of previous research

To the best of our knowledge, no previous trials have evaluated the effects of DCTs on similar outcomes. We therefore cannot directly compare our results to prior trials. However, as our program theory suggests that Komp may promote social connectedness, and thus prolong home-dwelling, we turn to studies on DCTs impact on subjective outcomes. As previously mentioned, only two full-scale RCTs have evaluated DCT as a standalone intervention. Czaja et al.’s [30] computer system (PRISM), designed specifically for older adults, was compared with Binder, a notebook containing paper content similar to that in PRISM. They found that PRISM significantly reduced loneliness and increased social support and showed tendencies to reduce social isolation after six months. These improvements, however, were not sustained at 12 months, despite continued use of PRISM, suggesting that PRISM’s static features limited opportunities for further engagement or skill development. Czaja et al.’s [31] follow-up trial found significant reductions in loneliness, social isolation, and improvements in social support, quality of life, and health-related quality of life. These improvements were only seen among participants in rural areas and senior housing locations – not for those residing in assisted living communities. The follow-up trial also included a mediation analysis, indicating that the reduction in social isolation and loneliness was a key pathway to improved quality of life. In sum, these trials indicate that DCTs may facilitate social connectedness in some contexts. Turning to interventions with clinical content, a meta-analysis of six RCTs on smartphone-based video calls and computer-based training aimed at reducing loneliness in older adults showed little to no effect [27].

Thus, the evidence for significant large-scale effects of DCTs remains limited. From an economic perspective, this raises questions about the long-term sustainability of widespread DCT implementation in health and social care. However, when viewing the evidence for DCTs improving social connectedness and quality of life [30, 31], in light of our findings of potential reductions in formal care use and associated cost savings, a more nuanced picture takes form. Specifically, the observed reduction in the duration of short-term institutional stays equals $1,500 per participant in a Norwegian context. However, the economic implications are uncertain, as we cannot say for certain whether differences in short-term stays were offset by transitions to long-term care facilities. Taken together, these findings suggest that while DCTs may hold promise as supportive tools in health and social care, the current knowledge base remains too weak to draw firm conclusions.

Our trial is the first RCT to evaluate the effect of a DCT on the use of formal care services among older adults. We have done so with a relatively large sample size, which proved necessary to detect any differences between groups, in a trial marked by non-compliance. Moreover, the use of high-quality registry data available from Statistics Norway allowed us to detect any skewness between groups on a broad range of variables with high accuracy. As our trial was conducted in a realistic municipal care setting within a conservative ITT approach, we have also enhanced the external validity and likely reduced the risk of unforeseen factors influencing the effects in a potential implementation at scale.

### Limitations

Our study has several limitations. First, the uptake of our intervention was low: of the 516 participants in the intervention group, 150 (29%) received a Komp device. This corresponds to 42% of those offered Komp (*n* = 359). Among those who received a device, 53 participants were registered for use. This corresponds to 10% of the entire intervention group, 15% of those offered Komp, and a third of those who accepted one. Among those who were registered with Komp use, the usage differed substantially with an average of 1.1 activities per week. Frequency does not necessarily reflect meaningful engagement, as previous research indicates that interactions on Komp often give a sense of everyday belonging, even though they are limited to events and do not occur daily [32]. However, compliance was lower than expected and lower than in other comparable trials [28, 30, 31]. As we follow an ITT-approach, the estimates should be interpreted as the effect of “belonging to the intervention group” rather than the isolated effect of the intervention [56]. While this reflects what could be expected in a large-scale implementation, it likely underestimates the potential positive or negative effects of the intervention itself.

Second, the statistical power of the trial was limited. Calculations showed that we would not likely be able to detect smaller effects, which means that the lack of significant findings in both the primary and secondary outcomes should be interpreted with caution, as meaningful effects may have gone undetected. Third, there is a potential SUTVA violation, as two control group members resided with participants in the intervention group who received a Komp device. One control group member was also mistakenly offered a Komp. Given the low number of contaminated participants, it is unlikely to have influenced the results. Fourth, there is always a risk of unobserved variables having influenced the results. Although access to comprehensive background data reduces endogeneity concerns by allowing adjustment for baseline differences in wealth and marital status, other relevant factors may have remained unobserved. For example, we did not have access to information on the number of household members, which could be indicative of access to informal care. If such support differed systematically between groups at baseline, this may have influenced the likelihood of remaining at home and the use of formal care. However, the baseline balance suggests that large systematic imbalances are unlikely. Similarly, we have no information on participants’ use of other DCTs, such as smartphones or tablets. If communication through other devices were already widespread, the marginal contribution of Komp may have been limited, and thus diluted the intervention’s estimated effect. Lastly, the generalizability of our findings may be limited. Previous trials have demonstrated that contextual factors matter, as a DCT proved effective only among older adults living outside assisted living communities [31]. Similarly, we must account for factors that restrict our findings to the context of our trial, e.g. that it was conducted in an urban area.

### Future research

We recommend that future studies continue to evaluate the effect of DCTs on formal care use in both similar and other contexts. These studies should also plan to investigate substitution effects between formal care services. Meanwhile, more trials assessing standalone DCT interventions, which are easier to implement at large scale, are needed. Additionally, it would be valuable for future research to explore more targeted approaches, as mentioned previously, which could both help prevent unintended harms and better reflect large-scale implementations. Further, we recommend evaluations of how Komp and other DCTs designed for individuals with less digital competence affect users’ social contact and well-being, to better understand the underlying mechanisms behind our findings. Finally, we recommend that researchers consider quasi-experimental approaches, such as propensity score matching, in trials with low compliance. In sum, more robust evaluations of DCTs across a broader range of outcomes are necessary to strengthen the evidence base for the digitalization of welfare states.

## Conclusion

In this trial, the results indicate that Komp, a digital communication technology, does not prolong home-dwelling among Norwegian older adults aged 67 years or older. The results further indicate that Komp reduces reliance on some formal care services, with short-term institutional stays reduced to an average of five days in the intervention group. We recommend further research to investigate how Komp affects users’ quality of life and well-being, to better understand the underlying mechanisms. We also recommend more robust evaluations of other types of digital communication technologies on various outcomes to strengthen the evidence base for the digitalization of welfare services.

## Supplementary Information

Supplementary Material 1.

## Data Availability

The data that support the findings of this study are not publicly available without further approval from Oslo Municipality and SIKT. The do-files can be accessed by contacting the authors. The trial is registered at ClinicalTrials.gov (NCT05919355). The trial protocol is publicly available [57].

## Abbreviations

OECD: Organization for Economic Co-operation and Development
DCT: Digital Communication Technology
RCT: Randomized Controlled Trial
PRISM: Personal Reminder Information and Social Management
ITT: Intention-to-Treat
CONSORT: Consolidated Standards of Reporting Trials
ADL: Activities of Daily Living
SD: Standard Deviation
HR: Hazard Ratio
SHR: Sub Hazard Ratio
ZINB: Zero-Inflated Negative Binomial
ZIP: Zero-Inflated Poisson
AIC: Akaike Information Criterion
BIC: Bayesian Information Criterion
IRR: Incidence Rate Ratio
OR: Odds Ratio
SUTVA: Stable Unit Treatment Value Assumption
